# Sequence and hierarchy in vocal rhythms and phonology

**DOI:** 10.1111/nyas.14215

**Published:** 2019-08-14

**Authors:** W. Tecumseh Fitch

**Affiliations:** ^1^ Department of Cognitive Biology University of Vienna Vienna Austria

**Keywords:** frame/content theory, rhythm, phonology, speech, evolution of language

## Abstract

I explore the neural and evolutionary origins of phonological hierarchy, building on Peter MacNeilage's frame/content model, which suggests that human speech evolved from primate nonvocal jaw oscillations, for example, lip smack displays, combined with phonation. Considerable recent data, reviewed here, support this proposition. I argue that the evolution of speech motor control required two independent components. The first, identified by MacNeilage, is the diversification of phonetic “content” within a simple sequential “frame,” and would be within reach of nonhuman primates, by simply intermittently activating phonation during lip smack displays. Such voicing control requires laryngeal control, hypothesized to necessitate direct corticomotor connections to the nucleus ambiguus. The second component, proposed here, involves imposing additional hierarchical rhythmic structure upon the “flat” control sequences typifying mammalian vocal tract oscillations and is required for the flexible combinatorial capacity observed in modern phonology. I hypothesize that phonological hierarchy resulted from a marriage of a preexisting capacity for sequential structure seen in other primates, with novel hierarchical motor control circuitry (potentially evolved in tool use and/or musical contexts). In turn, this phonological hierarchy paved the way for phrasal syntactic hierarchy. I support these arguments using comparative and neural data from nonhuman primates and birdsong.

## Introduction

The human capacity for speech has long attracted attention from linguists, anatomists, acousticians, and evolutionary biologists. Because certain fundamental aspects of the human speech capacity are absent in nonhuman primates, speech is a phylogenetically *derived* ability underlying human linguistic communication.^1^ Thus, its origins and subsequent evolution are one important component of any comprehensive model of language evolution.^2^ Other key components include our capacity for complex hierarchical syntax, along with certain aspects of compositional semantics and pragmatics.^3^

Three core computational components of language that provide its vast expressive power are combinatoriality (generating large sets from a limited set of building blocks), hierarchicality (tree‐like structure), and compositionality (structure‐based meaning). Together, these processes provide the “infinite use of finite means,” which provides an unlimited number of expressions that map to precise conceptual meanings. But these three processes clearly are related. In particular, the combination of tree‐like (syntactic) structures and word meanings yield the compositionality that provides unlimited possible meanings, while the combinatoriality of phonology combined with hierarchical structure yields the unlimited set of discrete expressions. Although it is tempting to assign each of these formal computational characteristics to a different module of language (combinatorial = phonology, hierarchical = syntax, and compositional = semantics), in this paper, I will pursue a different suggestion: that some degree of hierarchy is present in phonology (or music), independent of meaning, and that this provides a foundation for complex hierarchy as seen in syntax and semantics (both neurally and evolutionarily).

Returning to speech, my starting point for this argument will be the “frame/content” model of Peter MacNeilage and colleagues.^4^ MacNeilage has suggested that the core property of speech is the rapid alternation of the vocal tract between relatively open vowel states and relatively closed consonants (technically, obstruents). Together, this alternation provides the time‐varying oscillator that defines syllable structure and grounds abstract principles of phonology in the actual machinery of the vocal tract, providing building blocks fundamental to speech, including vowels, consonants, and syllables. The speech signal is more than just an unstructured, time‐varying pattern of formant frequencies with alternating phonatory states (e.g., voiced/unvoiced and tone), but has a flexible structure. MacNeilage has published considerably more on the topic,^5^, ^6^, ^7^ and until recently I felt I had nothing fundamental to add to his plausible story. However, recent data concerning speech rhythm in comparison with the physiology and neural basis of facial displays in nonhuman primates have led me to revisit the issue and the present review starts with an attempt to integrate these new data into the MacNeilage frame/content framework.

In general, I argue that MacNeilage's key evolutionary hypothesis—that speech rhythm is exapted from jaw oscillations involved in primate facial displays—is strongly supported by the available data and provides an important keystone in understanding the evolution of phonology. Recent studies have confirmed (as already argued by MacNeilage in 1998) that complex speech, fully capable of transmitting an arbitrary amount of linguistic information, can be produced using ordinary primate vocal anatomy. Thus, the much‐discussed descent of the human larynx was neither necessary nor sufficient for spoken language production. I next consider comparative data concerning the neural control of the vocal apparatus in order to isolate what, specifically, had to change in a generic primate brain to accomplish the key additional factor: accurate cortical control over voicing. This seems to involve a small but important innovation: direct (monosynaptic) connections from cortical neurons onto the motor neurons in the nucleus ambiguus that control phonation (specifically, the opening and closing of the glottal aperture). I review some suggestions about how these direct connections may have evolved and may be preserved ontogenetically, suggesting that this innovation was driven by selection for vocal imitation and song, as posited in Darwin's musical protolanguage hypothesis. This first part of the paper is essentially an update, using recent data and some tweaks, of MacNeilage's original hypothesis concerning the evolution of speech.

The second part of the paper explores some further implications of these ideas for other aspects of language, focusing on the crucial role of hierarchy in speech and phonology. Although vocal tract oscillations provide a crucial alternation of sounds, the resulting stream of protovowels and consonants has an essentially sequential structure, with no higher order structuring. Recent data from songbirds and human infant babbling indicate that such sequential structure is relatively “brittle,” in the sense that it does not allow free combination of arbitrary consonants and vowels, or of sequences of syllables: each specific combination must be laboriously built up via trial and error.^8^ I argue that the open‐ended combinatorial capacity of human phonology demanded more, requiring a system capable of imposing a flexible, nested hierarchical structure onto the sequential speech stream. Based on perceptual data from various nonhuman species, perceiving such a hierarchical structure is a challenge for other species, whose perceptual processing instead focuses heavily on sequential structure.^9^, ^10^, ^11^ However, recent motor data indicate that, with very extensive training, the beginnings of hierarchical structure are available to macaques in the manual/visual domain.^12^ I thus suggest that the acquisition of free combinatorics in phonology necessitated the imposition of hierarchical motor control onto the sequential structures generated by MacNeilage's “frame/content” mechanism. This vocal hierarchy, in turn, may have provided an exaptive precursor or preadaptation for the flexible hierarchical syntax that is central to all human language, whether spoken or otherwise.^13^ In other words, combinatoriality in phonology is based on a simple form of hierarchy, and this hierarchical phonology (also independently evidenced in music) provided a crucial preadaptive foundation for hierarchical syntax and semantic compositionality.

One possible source of selection for enhanced visual‐motor hierarchical capacities, still domain‐limited, would have been the ever‐increasing complexity of tool use and manufacture, well documented during hominin evolution. Skilled tool use and manufacture involves a network of brain regions including frontal (motor and planning), parietal (spatial), and temporal (memory of stored routines) with considerable overlap with those needed for spoken language.^14^, ^15^, ^16^, ^17^ I suggest that exuberant development of the pathways connecting these regions could have provided the initial basis for hierarchical motor control of the vocal pathway. Alternatively, the marriage of vocal sequencing with hierarchy that yielded speech rhythm may have occurred directly during the elaboration of protomusic from simple (birdlike) sequences to more complex song structures (as suggested for some songbirds). Although it is difficult to discriminate at present between these two plausible hypotheses, I provide some concrete testable predictions that might enable us to distinguish them in the future.

## Exaptation, evolutionary developmental biology, and the evolution of novelty

Some preliminary evolutionary points concerning the origins of novel neural circuitry are worth emphasizing. Darwin introduced the notion of “descent with modification” as a framework to conceptualize both similarities and differences among species, but tended to focus on the similarities in his discussions of human traits (combatting widespread assumptions of human uniqueness). By contrast, modern cladistic thinking in evolutionary biology (with its origins in systematics) emphasizes novel traits, particularly those that uniquely define a species or unify one clade relative to others (apomorphies and synapomorphies, respectively). When considering the origins of novel traits, we need to keep both perspectives in mind, since a novel trait (e.g., vocal learning) will be functionally advantageous only within a preexisting ecological, behavioral, and neural context. Adaptive apomorphies must thus be understood in their synapomorphic context.

I emphasize these issues because there is a tendency in discussions of language evolution to fall into one of two camps: “inclusivists” like Darwin who emphasized shared traits, or “exclusivists” like Descartes or Chomsky who highlight the unique traits. Any complete biological explanation involves a synergy between these two perspectives, and the ability of theorists to consider both aspects flexibly. Ultimately, speech is apomorphic to humans relative to other primates, and we need to first isolate and explain its derived features, and then understand their preexisting context, to understand speech evolution.

There is an increasing consensus that derived traits do not spring from nowhere fully formed, but instead arise from preexisting genetic material, developmental mechanisms, and neural circuitry. This means that virtually all known “innovations” have some precursors; these precursors are termed “preadaptations.” At the initial stage of change in function, the novel trait is termed an exaptation,^18^ but can later be fine‐tuned by selection and become a normal adaptation.^19^ Although genetic mutations are random with respect to their functional outcomes, they are strongly influenced and constrained by the past, and thus far from random when we consider the details of their mechanistic basis. One well‐known example is the phenomenon of gene duplication with later divergence, postulated to be a central mechanism in adaptive evolution by Susumu Ohno, and since then demonstrated in countless cases.^20^ For example, the phylogenetic origin of vertebrates involved two rounds of whole‐genome duplication (relative to closely related invertebrates), with subsequent loss or functional divergence of the copies.^21^, ^22^ This implies that to understand the evolution of a “novel” gene, we need to understand its precursor gene, role in development or physiology, and interactions with other genes. Most of this context will initially be shared by both the original and novel variant. Thus, it is unsatisfactory to “explain” the origin of some trait as a “mutation.” We want to know *what* mutation—which specific base pair or pairs—changed in what gene or allele.

Fortunately, modern evolutionary developmental biology (evo‐devo) has amply demonstrated that fundamental genetic and developmental processes are shared over large portions of the tree of life. In the 1980s, it initially came as a great surprise that precisely the same *Hox* genes play a key role in segmentation in flies and mice, or that the *Pax6* gene is expressed in the eye primordia in flies, fish, and squid. However, since then it has become clear that this is how evolution normally works: reusing components of an ancient, shared developmental genetic toolkit in amazingly diverse ways.^23^ In today's genomic and evo‐devo era, such evolutionary “tinkering”^24^ can now be understood in lovely mechanistic detail.

Turning to the brain, these evo‐devo principles can be integrated when considering the origin of new circuitry.^25^ First, the developmental processes by which vertebrate brains wire themselves up during development are broadly shared (e.g., between humans, frogs, fish, and mice), and when it comes to cortical regions and neocortical wiring and function, among all mammals. In vertebrates (though not all animals), the process of neural wiring is accomplished developmentally through cycles of exuberant growth (overconnection) followed by “pruning” of irrelevant connections,^26^, ^27^ a phenomenon that has been evocatively termed “neural Darwinism.”^28^ This means that organisms early in development will typically exhibit neural connections that are not seen (normally) in adult animals, a fact that can be used experimentally to “rewire” brains via early interventions. For example, the temporal cortex of ferrets normally subserves audition, but in ferrets whose occipital cortex and auditory thalamus are removed, the temporal cortex is rewired into a functional visual cortex.^29^, ^30^ Such developmental exuberance provides a ready source of “novel” connections during ontogeny, which under the right circumstances can be exapted into new circuits. “Novel” neural circuitry of the sort I discuss below is expected to rely on such broadly shared developmental processes and underlying circuitry, and to have precursors in related species.

Second, it seems likely that an analog of gene duplication can occur in the brain: neural regions and circuits can also duplicate and diverge. In particular, neural duplication has been postulated to play a key role in the origin of vocal learning abilities in birds and humans.^31^, ^32^ There are many other examples of existing circuitry being expanded and put to new use in comparative neuroscience. For example, the complex nasal appendages of the star‐nosed mole have the greatest mechanoreceptor density known, and allow phenomenally rapid identification and striking at prey, but represent hypertrophied exaptations of normal sensory skin around the nostrils.^33^, ^34^, ^35^ Weakly electric fish sense very weak electrical fields to locate prey or conspecifics, using their greatly enlarged cerebellum to “predict away” their own, self‐generated electrical field.^36^, ^37^ Despite its evolutionary novelty, this relies on the same delay‐line cerebellar architecture found in all vertebrates.^38^ Taken together, these two principles—exuberance for rewiring, and duplication with divergence—provide a powerful exaptive framework for understanding brain evolution. But for both, a clear understanding of the initial preadaptive state is a central requirement.

## Vocal learning as a key innovation for speech

I now discuss what I see as a key derived aspect of human speech: complex vocal learning. By this I mean the capacity to learn to produce sounds heard in the environment that are outside the innate vocal repertoire (by “innate,” I simply mean reliably developing, species‐typical, with no implication of “fixed” or involuntary^39^). Considerable discussion in the last decade concerning vocal learning exhibits a rising tendency to reject earlier discussions emphasizing the dichotomy between vocal learners (like humans) and nonvocal learners (like chimpanzees). Several scholars have suggested that in fact there is a continuum between these, and emphasized that nonhuman primates (“primates” hereafter) do in fact have some vocal learning abilities (e.g., Refs. 40, 41, 42). I argue that this rising tide is simply muddying the conceptual waters in two ways: they fail to adopt clear and widely accepted terminology delineating different categories of vocal learning;^43^, ^44^ and they fail to acknowledge decades‐old data showing several types of “vocal learning” in virtually all birds and mammals tested.

Because the data concerning vocal learning have been frequently reviewed recently, I will discuss only the broad conclusions and not their detailed empirical basis here; for details see Refs. 43, 45, and 46, and a recent journal issue that provides concise overviews of complex vocal learning in all of the currently known clades.^47^ The first step in discussing vocal learning is to adopt a clear terminology, because there are many types of vocal learning, with very different phylogenetic distributions. The field today largely follows definitions of three distinct forms of vocal learning, laid out by Janik and Slater,^43^, ^44^ as I do here. First, many species can learn the meaning of novel sounds (including heterospecific sounds like bird alarm calls or human speech); Janik and Slater term this “comprehension learning.” Furthermore, virtually all birds and mammals tested can learn to voluntarily produce or inhibit their species‐typical vocalizations, so‐called “usage learning.” Comprehension and usage learning are together termed “contextual learning,” or learning *about* sounds, and are both very widespread in birds and mammals, but neither constitute “vocal learning” (or more precisely “vocal production learning”) in Janik and Slater's terms. They use this term to denote that “vocalizations are modified in form as a result of experience with those of other individuals.”

However, as pointed out more recently by Tyack,^46^ even this strict definition allows many distinct types of modification. For example, the Lombard effect is the phenomenon whereby vocalizers increase the intensity of calling in the presence of noise, and is ubiquitous among mammals and documented in both frogs and grasshoppers.^46^, ^48^ If this online intensity change occurs in response to conspecific vocalizations, strictly speaking, it constitutes “vocal production learning” according to the above definition. Similarly, males that increase the pitch or intensity of an innate mating call when hearing a female vocalize might be classed as “vocal learning” by their definition. But a commonsense interpretation of “learning” would not typically consider such online, reflexive modifications as “learning.”

Similarly, there is considerable evidence across taxa, including primates, for “vocal convergence,” meaning that the vocalizations of individuals in pairs or groups become more similar over time (cf. Tyack^46^). Although this may in some cases involve learning something novel (e.g., in budgerigars^49^) it might also simply reflect changes in arousal or other socially mediated factors. For example, a much‐discussed convergence in chimpanzee food grunts given when seeing apples by a newly introduced set of individuals was described as vocal learning in the original publication.^50^ This interpretation was convincingly challenged in a follow‐up paper,^51^ which suggested that a simple arousal‐based explanation (the new animals eventually became bored by apples) was not adequately rejected. It is thus crucial to separate learning a novel call type from modifying a call in the existing vocal repertoire.

Such considerations have led me, and many others, to draw a finer distinction than Janik and Slater's original, and to define “complex vocal learning” as the ability to acquire novel vocalizations, outside the biologically given species‐typical repertoire. The most convincing evidence for complex vocal learning is “vocal mimicry,” when animals learn to imitate heterospecific sounds (such as human speech or song), an ability currently documented in parrots, various songbirds, cetaceans, harbor seals, and both elephant species (and of course humans). However, the acquisition by one individual of sounds produced by a conspecific model that are NOT part of the species‐typical repertoire (i.e., sounds that do not reliably develop in the absence of the model sounds) is equally convincing evidence of complex vocal learning, and adds various hummingbird and bat species to this list (along with many other bird and cetacean species). By these two criteria, complex vocal learning (“vocal learning” hereafter) is currently known in the following clades: hummingbirds, parrots, songbirds, cetaceans, pinnipeds, bats, elephants, and humans. Although the evidence in some of these cases is stronger than others, and it is likely that other vocal learning species exist but are not currently documented, this relatively short list is what most contemporary scholars (some primatologists excepted) consider to be “vocal learning” species.

Let us turn now to two controversial clades: primates and rodents. Based on an impressionistic similarity between mouse ultrasonic courtship vocalizations (when slowed down to an appropriate replay speed) and birdsong, Holy and Guo termed these vocalizations “mouse courtship song” and raised the question of “whether mice, like birds, learn their songs through experience.”^52^ Given the ubiquity of the laboratory mouse as a model organism, and some suggestive changes in vocalizations in “humanized” mice bearing a human *FOXP2* gene,^53^ this led to a flurry of discussion concerning possible mouse vocal learning.^31^ In my opinion, several recent studies convincingly reject this possibility: deaf male mice produce a normal song and song differences between cross‐fostered mouse breeds reflect biological origin and not the rearing environment.^54^, ^55^, ^56^ Thus, mice appear, like most mammals, and birds other than parrots, songbirds, and hummingbirds, to lack a capacity for complex vocal learning (cf. Tyack^46^).

Turning to primates, although human vocal learning is unquestioned, and central to both spoken language and song, repeated attempts to teach nonhuman primates to speak or sing, or even produce very simple novel vocalizations, have universally failed. This includes repeated experiments where infant apes were raised in human homes.^57^, ^58^ This inability contrasts very clearly with the abundant positive evidence for both intentional control over species‐typical vocalizations or online phenomena like the Lombard effect (seen in all primates tested), or vocal convergence (documented in multiple species). A recent study claiming that an orangutan matched the pitch of a novel vocalization to that of a human model is unconvincing:^40^ the behavior did not involve matching of vocalizations, the claimed correlation in pitch was very weak, and the authors did not exclude the possibility that the human (who could clearly see the orangutan) was intuitively reading the ape's mood and matching the ape, rather than vice versa.

Thus, abundant data collected over many decades reveal voluntary control over vocalization, and a capacity for call convergence in existing calls, but no complex vocal learning in primates. There are, however, two interesting cases that appear, initially, to defy this clear pattern. The first is the control and apparent learning of some nonphonated sounds (lip buzzes or “raspberries”) by chimpanzees,^59^ and the clearly documented lip whistling by a single orangutan.^60^ There is no evidence that this orangutan acquired whistling from human models (she may have spontaneously invented it). However, there is clear evidence for a cultural spread across a group in the chimpanzee case.^61^ Finally, the enculturated gorilla Koko shows clear voluntary control over her breathing, making a variety of huffs, lip buzzes, and even playing the harmonica.^62^, ^63^ What makes these apparent exceptions interesting is that none involves control of phonation, but rather of the jaws, lips, and tongue (and some respiratory control)—a fact that will become crucial below when we consider mechanistic hypotheses about the neural basis of vocal learning.

### Changes in vocal anatomy are neither necessary nor sufficient for vocal learning

One long‐standing red herring concerning the mechanistic reasons for the absence of complex vocal learning in primates can now hopefully be laid to rest: the notion that the descent of the human larynx (or other more minor changes in peripheral vocal anatomy) were needed to confer vocal learning on our species. Although Darwin discussed the idea that vocal anatomy might be a key limitation on vocal learning in primates, he considered any anatomical changes less important than changes in neural control.^64^ But anatomical changes have been repeatedly suggested since then to be crucial for the human power of speech.^65^, ^66^, ^67^, ^68^, ^69^ The most convincing possibility, suggested by Philip Lieberman and colleagues, was that the lowering of the human larynx and resulting reconfiguration of the human vocal tract makes a range of sounds possible (particularly the “point vowels” /i/, /a/, and /u/ of “beet,” “bought,” and “boot,” respectively) that would otherwise be impossible to produce with a nonhuman primate vocal tract (which is *not* the same as claiming that these anatomical changes are necessary for complex vocal learning). But even this more modest hypothesis has been convincingly challenged based on computer models and data from nonhuman primate vocalizations,^70^, ^71^ along with X‐ray observations showing that all mammals studied spontaneously lower the larynx during vocalization.^72^

My colleagues and I recently reexamined this issue by capturing X‐ray videos of macaques vocalizing and producing other vocal tract movements (facial gestures, such as yawns and lip smacks, along with chewing and swallowing). We used these to build a computer model strictly limited to those vocal configurations that were actually observed in a single macaque.^73^ We then calculated five optimal vowels for that simulated vocal tract and played them to human listeners: they were easily able to discriminate all five at levels comparable to those observed for human vowels. Finally, we used our model to produce entire sentences; these again were readily understood by human listeners. Thus, macaque vocal tracts are “speech ready” in the sense that they could produce intelligible speech without any anatomical modifications whatsoever. The complete lack of anything like speech in nonhuman primates cannot be attributed to their vocal anatomy, a conclusion with which Lieberman concurs (who called our study, to the surprise of many readers, a “replication” of his earlier work, cf. Refs. 74 and 75).

I conclude, as emphasized by Darwin, that there is a fundamental difference between vocal learning abilities in humans and our nearest primate relatives. The human capacity for complex vocal learning represents an important derived feature of our species that we need to explain in neural, developmental, and ultimately genetic terms, and is not a result of changes in human vocal anatomy such as the descent of the larynx.

### Direct connections as a key neural basis for vocal learning

I now turn to the neural and developmental basis of this ability, starting with a brief description of the complex neural control of the vocal apparatus. Most of the motor neurons that innervate vocal musculature are in the brain stem, with a few in the spinal column involved in respiration. These “motoneurons” send axons via the cranial and spinal nerves to synapse on their final muscle targets, including at least 26 separate vocal muscles.^76^ The relevant cranial nerves include the hypoglossal nerve (to the muscles of the tongue), the facial nerve (lips), the trigeminal nerve (jaws), and the vagus nerve, innervating the larynx and some respiratory muscles.

The current leading hypothesis explaining the increase in vocal control necessary for human speech has been called the “direct connections” hypothesis, or the “Kuypers/Jürgens” hypothesis after its originators.^77^, ^78^ This hypothesis holds that accurate voluntary control over musculature requires direct monosynaptic connections from cortical motor neurons to the final brain stem motoneurons described above. The idea that voluntary control requires such direct connections is well established in the domain of hand and limb control, based on detailed comparisons between mammals, including primates, who possess or lack such connections.^79^, ^80^ Mammals, such as cats or squirrel monkeys, who lack direct connections to the spinal motoneurons innervating finger muscles are unable to independently control their fingers, whereas those species possessing direct connections (e.g., capuchins, chimpanzees, or humans) have fine finger control.

Applied to the vocal domain, there is a considerable variation in the degree to which direct connections are present to the different vocal motor neurons, meticulously documented in primates by many tracing studies by Jürgens and colleagues,^77^, ^81^, ^82^, ^83^, ^84^ along with some earlier and less reliable data from chimpanzees collected by Kuypers.^78^, ^85^ The crucial observation emerging from this mass of data is that many primates, including apes, have direct connections to the muscles controlling lip, tongue, and jaw movements (the vocal tract) but *not* to laryngeal motoneurons, located in the nucleus ambiguus, in the medulla. In humans, both tracing and electrical stimulation studies show that direct connections are present from the cortex to all of these neurons, including all the laryngeal motor neurons.^1^, ^86^

In chimpanzees but no other studied primates, there appear to be sparse connections to the nucleus ambiguus, but these only reach the anterior‐most portion of this nucleus, which innervates the cricothyroid muscle (crucial for pitch control) and not the posterior portion innervating the other intrinsic laryngeal muscles like the cricoarytenoid muscles that are required for initiating phonation.^87^ Although the situation in other apes is unknown, assuming a similar sparse presence of direct connections to the anterior‐most ambiguus motoneurons would allow apes an intermediate level of fine laryngeal control, relative to humans, but not the rapid on‐and‐off alternation of voiced and unvoiced segments observed in human speech.

This pattern of innervation is precisely consistent with the apparent examples of ape “vocal learning” discussed above: both lip buzzes and whistling require control over the lips, jaws, tongue, and respiration (present in apes) but *not* over the initiation or inhibition of phonation (which, based on the connectivity pattern, should be absent in chimpanzees). Thus, these examples, in fact, constitute exceptions that prove the rule, providing additional support for the direct connections hypothesis.^1^ Furthermore, turning to the other vocal learning species discussed above, direct connections have been documented in all three clades of vocal learning birds,^88^ and are currently being investigated in vocal learning bats. Thus, a considerable amount of data from various species and methods, all provide convincing support for the notion that direct corticomotor connections, specifically onto the motoneurons in the posterior nucleus ambiguus that support phonatory control over voicing, represent a derived feature of humans relative to other primates and play an important role in our complex vocal learning abilities.

A skeptic might reply that the larynx is important for singing, where very fine laryngeal control is necessary to sing in tune^89^ but is not crucial for speech. Laryngectomy patients can communicate with a simple buzzer held to their neck, and whispering involves no voicing at all (but rather a simple laryngeal constriction to generate noise). But in both cases, it is only redundant lexical context that allows a trained listener to understand the speech. Furthermore, very rapid control of voicing is central to the voiced/unvoiced distinction made in all languages (with voice onset time differences on the order of 100 ms determining completely different consonants like /p/ and /b/90). Finally, the majority of the world's languages use fine variations in tone pattern phonemically,^91^, ^92^ again requiring fine control over the larynx.

It is worth noting that, in addition to such direct connections, other corticocortical connections that may play a key role in vocal learning seem to be greatly enhanced in humans relative to other primates. In particular, the arcuate fasciculus connecting temporal, parietal, and frontomotor cortical regions is greatly developed in humans relative to chimpanzees, and extremely limited in macaques.^93^, ^94^ Because complex vocal learning appears to require comparison of auditory templates with vocal output during ontogeny (see below), connections from the auditory (temporal) to the motor (frontal) cortex are probably particularly crucial. Thus, the hypertrophy of these cortical pathways is probably another derived feature relevant to human vocal learning.^1^

### Developmental origins: babbling and neural Darwinism

I now turn to the ontogeny of direct connections. Beautiful developmental data from songbirds indicate that changes in the expression of cadherin molecules during development play a central role in the establishment of neural connections in the song system.^95^, ^96^ There is currently no comparable data on the genetic basis for direct connections in humans or other mammals, but bats may turn out to provide an excellent model species to resolve this issue.^97^ Thus, the genetic underpinnings of direct connection formation remain uncertain at present.

There is, however, a tantalizing developmental hypothesis about the potential role of babbling in supporting or preserving direct connections. *Babbling*, or vocal play during the period of vocal learning, is well documented in humans as well as songbirds and vocal‐learning bats and has been suggested in cetaceans and elephants.^8^, ^98^, ^99^, ^100^, ^101^, ^102^ Babbling appears to constitute an arena in which trial‐and‐error matching of immature vocalizations to stored auditory templates can occur. Babbling is self‐reinforcing in humans and birds: there is an intrinsic drive of young animals to babble. This leads me to suggest that babbling is in fact a prerequisite for complex vocal learning, a proposal testable by examining ontogeny in species in which it has not yet been documented (e.g., hummingbirds, seals, or cetaceans).

But there is a deeper evo‐devo reason that babbling may be necessary for vocal learning, concerning direct connections. Terrence Deacon^103^ suggested that, during development, transitory direct connections may be formed in many mammals between cortical neurons and cranial motoneurons, but that these transient connections are outcompeted during development by rival connections from the ancient circuitry subserving innate vocalizations (particularly projections from the periaqueductal gray in the midbrain). Deacon suggested that increases in forebrain size (augmenting the cortical connections) could change the bias in this competitive process, allowing direct connections to persist as a side effect of brain expansion. Another plausible factor in this tipping of the competitive balance might be a reduction in the innate call repertoire that Deacon suggests occurred during hominin evolution, which could weaken the midbrain projections.

But the most obvious possibility is that the behavior of babbling itself biases this neural Darwinistic process in favor of the cortex (see section 10.4 in Ref. 86). By setting up self‐rewarding Hebbian reinforcement of direct corticomotor connections, the behavior of babbling may bias the cortical control system to preserve direct corticomotor connections at the expense of the more ancient and prepotent connections from the midbrain. A set of tantalizing anecdotal observations of Viki, a young chimpanzee raised in a human household, is consistent with this babbling/direct connections hypothesis.^58^ One day, Viki's caretaker Catherine Hayes noticed that she “went Hawaiian” and began, unprompted, to make sounds reminiscent of human babbling. This was at the age of about 4 months, just before babbling takes off in human infants. However, almost as soon as it began, this behavior ceased forever.

This anecdotal observation suggests that babbling‐like behavior might have been present at some low level in the common ancestor of humans and chimpanzees, and the simple expedient of making babbling behavior more intrinsically rewarding might have motivated early infant hominins to babble more persistently. If Deacon's neural Darwinism idea about the competition of rival neuron populations is correct, such a change in motivation might have played a key role (or have been fully sufficient) to drive the Hebbian preservation of direct connections in the hominin lineage. Although this is speculative, it is consistent with recent data from songbirds demonstrating a key role for dopaminergic reward circuitry, via projections to song nuclei, in the acquisition (by listening to singing adults) of the auditory template needed for the young bird to learn its song.^104^, ^105^

## The origins of phonological streams

Having established the importance of complex vocal learning as a derived component of the human speech capacity, I now turn to my second main question: what additions were required for an organism with vocal learning abilities to gain the free combinatorial abilities over consonants, vowels, and syllables that are observed in human spoken language? Vocal learning by itself gives an organism phonetic control, but phonological constructs require additional, higher order combinatorial abilities as well. My argument here starts by recapping MacNeilage's frame/content theory (FCT) and reviewing strong recent empirical support for his ideas. I suggest that this theory can explain the origin of variegated sequential structure. However, the hierarchical structure of phonology is not accounted for and will be my focus below.

### MacNeilage's frame/content theory

Peter MacNeilage developed his FCT over several decades, and it has detailed phylogenetic, mechanistic, and developmental components. Given its scope, I can only review highlights here, and I recommend his 2008 book^5^ for more detailed arguments (for pros and cons, see also section 10.3 of Ref. 86). The overarching idea is that the speech stream is made up of two components. The first is a periodic syllabic framework (at roughly 5 Hz), controlled by a medial motor system centered on the supplementary motor areas (SMA and pre‐SMA). The second is the phonetic “content:” the individual vowels and consonants that “fill” syllabic frames. This component is hypothesized to rely upon the “classical” language areas in the lateral peri‐Sylvian motor system (centered on Broca's area). Thus, frame and content rely on these different neural control systems and have somewhat independent evolutionary histories.

For my purposes here, FCT makes two main claims. The first claim is phylogenetic: that the origin of syllable streams in speech should be sought not in primate vocalizations, but in nonvocal facial displays, particularly the lip and jaw oscillations observed during lip smacks and related displays that are common in catarrhine primates (Old World monkeys and apes^106^). The second claim is mechanistic: that the syllabic frame that provides the rhythmic basis for the speech stream is generated by the medial motor system, especially SMA. This claim is based on abundant brain stimulation and brain lesion data in humans, summarized already by MacNeilage and colleagues.^5^, ^107^ The key consistent finding is that activation of the SMA yields repeated phonetically well‐formed syllable streams (“ta ta ta,” “eh eh eh,” and the like). This is unique: stimulation of Broca's area (or basal ganglia) can inhibit or halt speech, but not generate it. It also occurs not only with (artificial) electrical stimulation but with irritative lesions that generate hyperactivity in SMA. Given its consistency, this finding has gone relatively unappreciated in contemporary neurolinguistics; part of the reason may be that SMA may share this “framing” role with other aspects of complex motor activity. If so, the logic of the subtractive paradigms often used in brain imaging would often exclude SMA activity, since it is not speech specific.

### Behavioral data strongly support MacNeilage's phylogenetic claim

My initial appraisal of the phylogenetic component of FCT was somewhat skeptical: although it is true that jaw oscillations are seen in lip‐smacking, they are also seen in vocalization sequences, providing little reason to favor one over the other. Also as I noted in Ref. 86, speech is almost unique among mammal vocalization in featuring extreme tongue movements as well as jaw oscillations, and “lip‐smacking” did not seem relevant to this key‐derived feature.

My appraisal changed sharply when, together with Ghazanfar *et al*.,^108^ I observed real‐time X‐ray video of lip‐smacking in macaques. I have spent considerable time acquiring and analyzing X‐ray video of animal vocalizations and human speech, and the first thing that jumped out when observing macaque lip‐smacking was the extreme and highly synchronized *tongue* movements accompanying the lip and jaw movements. Although lip‐smacking in macaques is unaccompanied by phonation, the X‐rays gave an immediate and strongly speech‐like impression. Although these tongue movements are partially visible externally as tongue tip protrusions from the lips (particularly in retrospect, with slow replays of high‐speed video), it was only with X‐ray imaging that these highly synchronized lip, jaw, and tongue movements become clear. We used detailed analysis^108^ to show both that macaque lip smacks involve the same articulators as speech (tongue, jaw, and lips), occurring at essentially speech rates (5−6 Hz), and that lip‐smacking resembles speech much more than chewing (the much slower oscillations of which we also analyzed). We concluded that these X‐ray lip‐smacking data provide strong support for FCT. The tight and stereotypic coupling between tongue and jaw movements (which is looser during the variegated syllables making up speech) indicates that lip‐smacking is relevant only for the frame component (as hypothesized by MacNeilage). For a more detailed review of these and other relevant data, see Ref. 109.

Intriguingly, there is one apparent example of a vocalized lip‐smacking sequence: the “wobble” call of the gelada baboon *Theropithecus gelada*.^110^ These are prolonged vocalizations, produced mainly in affiliative contexts by males toward females, consisting essentially of a “normal” lip‐smacking sequence, coupled with clear voicing (by itself termed a “moan”). Unlike speech, wobbles are consistently voiced throughout, with no alternation of voiced and unvoiced components, but nonetheless they represent a clear intermediate case, and thus proof‐of‐concept, that voiced lip‐smacking can evolve and be stable (they are not present in other closely related baboon species). This also indicates that neural studies on geladas would be extremely valuable in uncovering the neural bases required for controlled voicing in speech.^109^

### Support for MacNeilage's mechanistic claim is equivocal

Regarding neural control, new data are only partially consistent with FCT. Most of the animal work is in the context of the macaque mirror‐neuron system, and it is clear that mirror neurons exist that are both active during lip‐smacking, and activated by observing lip smacks.^111^ It is less clear that such neurons are specific to SMA as predicted by the FCT; the existing data report them only from the lateral cortex. There are no studies reporting stimulation of SMA in monkeys yielding lip‐smacking (but in both cases, missing data are not evidence of absence). The finding that motor neurons are activated during observation of lip‐smacking sequences is certainly compatible with the broader suggestion of FCT that the “syllabic” frame of lip‐smacking has both motor and perceptual components. Also, the timing of neural activity to lip‐smacking and its synchronization at syllabic rates around 6 Hz is consistent with FCT. What is more, monkeys show a preference for computer‐animated lip smacks at this speech rate,^112^, ^113^ additional evidence for perceptual “tuning” to these displays. Similar rates of vocal modulation have been recently shown in multiple primate vocalizations.^109^, ^110^, ^114^, ^115^

An important recent paper used functional magnetic resonance imaging to examine brain activation in macaques who were induced to produce lip smacks by showing them videos of conspecific faces.^116^ The production of these communicative facial gestures activated *both* medial and lateral motor areas, though with a bias toward the medial regions. This finding is consistent with an overall framework of medial and lateral areas being involved in both lip‐smacking and speech, but contrasts with the basic mechanistic prediction of the FCT that medial areas alone should be active in generating “syllabic frames.” However, now that we know that some tongue‐based “content” is also present in macaque lip‐smacking, I consider these new data consistent with the FCT's phylogenetic claim of lip‐smacking as a speech precursor. In fact, the finding that lateral motor regions are involved as well makes the phylogenetic aspects of this idea *more* consistent. But it does raise the question of what else needed to happen, in terms of neural control, to yield the variegated, freely combinatorial syllable sequences of human speech from the stereotyped and reduplicative sequences seen in lip‐smacking. I turn to this question below.

I conclude that, in addition to the considerable amount of data already cited by MacNeilage in support of FCT, newer physiological and neural data are all consistent with the fundamental predictions of his hypothesis. To evolve a speech stream, all that is required is (1) lip‐smacking (an Old World primate synapomorphy), plus (2) voicing (as in geladas), and plus (3) complex vocal learning for phonatory control. For the rest of this paper, I provisionally accept both the phylogenetic and mechanistic framework of FCT, updated to include lateral cortical activity during lip‐smacking, and ask “What else is needed for modern human speech?” Again, new animal data hold some surprises.

## Origins of phonological hierarchy

Thus far, I hope to have established the following propositions. First, anatomical changes were neither necessary nor sufficient for the evolution of human speech; instead, the synapomorphic vocal anatomy of the ancestral primate would have been perfectly adequate. Second, a key derived feature needed to evolve vocal learning in speech was the presence of direct corticomotor connections, specifically from the laryngeal motor cortex onto the motoneurons in the posterior nucleus ambiguus, that control phonation onset and offset. Third, Peter MacNeilage's frame−content theory posits a phylogenetic precursor for speech movements in the lip‐smacking gestures of catarrhine primates, and this hypothesis is well supported by behavioral and neural data. However, even in lip‐smacking, the lateral motor cortical regions (analogs of Broca's area) are already active (in addition to the medial regions predicted by FCT). It thus led to my final question: What other changes in these neural control regions were needed to yield the capacity for human speech? A recent birdsong study offers surprising new clues to help answer this question.

### Variegated sequences do not yield free combinatoriality

The workhorse species for neural investigations of birdsong is the zebra finch *Taeniopygia guttata*, a species chosen not for the beauty or complexity of its song, but for ease of breeding in the laboratory. In fact, zebra finch songs are nasal and highly repetitive, and a given adult male sings a fixed song for the rest of his life: they hardly represent the epitome of beauty or flexibility in birdsong. However, like most songbirds, this “crystalized” adult song is preceded by a more variable subsong stage, reminiscent of human infant babbling, in which the bird appears to be gradually acquiring its adult song through a trial‐and‐error process.^117^ With careful timing, this acquisition process can be interrupted in midstream by providing a new auditory template to the learner, inducing it in some cases to adopt the new song.

In one of the most surprising findings of the last decade, Lipkind *et al*.^8^ performed a clever manipulation using this approach. They first trained a young male on some recorded sequence ABC, but then, during his subsong stage, switched this template to BAC (where the letters represent particular song “syllables” or units). If zebra finches had a freely combinatorial system, this would be trivial to imitate (because the bird could already produce A, B, and C syllables) and the switch to the new song should occur readily. On the other hand, if birds must additionally learn the *transitions* between syllables, the new song would be a challenge (since neither the BA nor AC transitions were initially present). It turned out that this task was a real challenge, and only some birds succeeded. Surprisingly, successful birds had to laboriously acquire each of these new transitions piecemeal. This demonstrates a lack of free combinatoriality in zebra finch song learning.

Given the simple nature of zebra finch song, this might not generalize to other species. The researchers thus examined the acquisition of syllable transitions in Bengalese finch song (which is more complex and flexible) and in human infant babbling. In both cases, the same pattern was found: each new syllable type gradually acquired a diversity of pairwise transitions, in a stepwise fashion during development. That is, even human infants do not immediately transition from repetitive “reduplicated” babbling (“dadada” or “gagaga”) to variegated babbling, where all combinations of these syllables are equally possible. Instead, they had to acquire the “daga” and “gada” transitions separately.^8^

Thus, our adult ability to readily repeat nonsense words, whatever their sequential structure, as long as they are consistent with the phonology of our language(s), came at a cost. These data demonstrate that during infancy we had to actually build up the entire transition matrix that underlies this free recombination ability, transition by transition. Obviously, without such recombination, the phonological system would be extremely limited, and would not easily yield the vast possible phonological vocabulary needed to transmit arbitrary meanings in language. Although “da da da” sequences might be adequate for conveying melodies in some protomusic, they cannot suffice to generate the large vocabulary needed for complex propositional meanings in language. Thus, the achievement of free combinatoriality is surprisingly challenging, and understanding the evolution of open‐ended phonological capacity in humans requires an explanation of this ability, in mechanistic and evolutionary terms.

### Whence free combinatoriality? Fixed‐depth hierarchy as the middle term

Language has long been recognized to represent a hierarchical system, where a finite set of small parts (like segments, syllables, and words) are combined flexibly to yield essentially infinite possible outputs. Although this is traditionally emphasized in phrasal syntax, it is equally true of phonology.^118^ To give an impression of this “infinite use of finite means” in phonology, consider a language simpler than English that has 10 vowels (Vs) and 10 consonants (Cs) that can be arranged freely into simple CVC syllables. This would yield 10 × 10 × 10 or 1000 possible syllable types. When these are combined into four‐syllable words, this yields 1000^4^ (= 10^12^) or 1 trillion possible words, vastly more than are used in English or any other language. Although not infinite, this shows that phonology has more generative potential than necessary for communication—but only if free combination of segments into syllables and syllables into words is possible. Of course, in natural language, there are some restrictions on combinations, termed phonotactic constraints, but these do not greatly reduce the combinatoric potential. This is why there are a huge number of unused “possible words” or pseudowords in any language (“wuggish,” “biffulated,” and “slombulant” are random examples of pseudowords that obey English phonology but are not part of the lexicon).

Turning to the domain of phrasal syntax, the recombination of thousands of words into phrases and those phrases into sentences yields an even greater combinatorial explosion. To the extent that there is no fixed limit on depth of embedding in syntax (since such structures as “John's girlfriend's father's car's carburetor,” or “John thought his girlfriend told her father that his car's carburetor is broken” are perfectly acceptable), this allows a potentially unlimited number of sentences to be generated, each with a particular discrete meaning. One can of course point to real‐world limitations (on breath, memory, or patience) that prevent phrasal syntax from being infinite, but nonetheless the number of possible sentences vastly exceeds the number of possible meaningful thoughts one might want to express in a lifetime, and it is this excess generative capacity (not “infinity”) that is a design feature crucial for language.

These two levels of hierarchy in language together provide the so‐called “duality of patterning” in which meaningless segments and syllables can be combined to form meaningful words or morphemes (phonology) and meaningful words/morphemes combined to form sentences (syntax).^119^ The key difference between the two levels is that in phonology there is a *fixed depth* to the hierarchy because there is no self‐embedding (you can embed segments into syllables, or syllables in words, but you cannot embed a syllable within a syllable), rendering its combinatorial capacity large, but finite. By contrast, the recursive phrasal syntax allows self‐embedding (phrases within phrases), and therefore a theoretically unlimited number of distinct sentences. Although the recursive aspect of syntax has received a huge amount of attention,^120^, ^121^, ^122^, ^123^, ^124^, ^125^, ^126^ the origins of the fixed‐depth hierarchy needed for phonology has received less consideration.

Perhaps, this neglect is due to an unspoken assumption that, once you have segments, you can freely combine them into syllables, and once you have syllables you can freely combine them into words. But the birdsong and infant babbling data just discussed strongly suggest that this is not the case—recombination does not “fall out for free” from a simple set of segments or syllables. Put in MacNeilage's terms, even with a syllabic frame and some segmental content explained, we still need to understand where the flexible combinatoric capacity of phonology came from. Indeed, the very term “hierarchy” connotes some flexibility in the components that are hierarchically combined. This means that we can also frame this question in terms of the origins of phonological hierarchy.

### Potential preadaptive origins of combinatoriality

My phylogenetic suggestion here is that because fixed‐depth hierarchy is less computationally challenging than open‐ended embedding, the required circuitry could evolve by duplication and divergence of brain mechanisms that were already available to our primate ancestors. I, therefore, suggest that fixed‐depth hierarchy thus provides an evolutionary middle term between simple speech sequences and complex hierarchy. This fixed‐depth hierarchy was achieved first in phylogeny in the same way it is achieved, laboriously, during ontogeny in birdsong or babbling.

Like MacNeilage, my mechanistic hypothesis relies on the recruitment of the lateral motor system (Broca's area and its connections in parietal and temporal cortices). In MacNeilage's model, this lateral system is responsible for programming the “content” inserted into the syllabic “frame” generated by the medial system. Although I agree, I think the precise computational role of this content specification needs to be further elaborated. Specifically, I suggest that Broca's area plays the computational role of a limited‐depth “stack:” essentially a memory buffer (or variable) that contains, at any moment, the syllable identities making up the current phonological word. Each syllable identity, coded by activity in Broca's area, corresponds to the stored motor programs for a fixed series of segments that themselves are coded in the motor cortex and recalled from the temporal cortex. Thus, Broca's area acts as a “clearing house,” one level of the phonological hierarchy up from segments and syllables. Representations of different levels of the phonological hierarchy are distributed in different brain regions, with the memory buffer in Broca's area pulling them together into a phonological word.

My second suggestion is that this simple fixed‐level hierarchy itself inherited from other preadaptive neural circuitry. There are at least two plausible precursors. The first (related to that specified by MacNeilage, as well as some suggestions of Lashley^127^, ^128^) is that toolmaking and tool‐use circuitry would have been ripe for exaptation as the capacity for vocal control evolved. Starting from the already relatively sophisticated tool use present in our last common ancestor with chimpanzees,^86^ toolmaking and tool use had been steadily advancing in sophistication in early hominins, providing a hierarchical structuring control system that originated in tool use, but “ready‐made” for exaptation into vocal usage. This hypothesis is clearly consistent with the archaeological record, in the sense that even very early hominins (the australopithecines) had already advanced considerably over chimpanzees in the toolmaking and presumably tool‐use behavior;^14^, ^15^ its predictions can be tested by evaluating and comparing the neural underpinnings of speech and toolmaking.^16^, ^17^

The second possible precursor is more speculative: that the circuits put to use in the phonological hierarchy in fact evolved in the vocal domain, but for use in song rather than speech. This hypothesis is based on the assumption of a “musical protolanguage” as hypothesized by Darwin.^64^, ^129^ A *protolanguage* is a hypothetical construct, representing an evolutionary stage on the way to modern human language that possesses some but not all of the features of modern language.^130^ The term was introduced in the context of “gestural protolanguage” by Hewes,^131^ and later popularized to indicate a “lexical protolanguage” (or system with meaningful words, but lacking syntax) by Bickerton.^132^ By contrast, recognizing the importance of vocal learning, Darwin suggested that initial function of vocal learning was in the production of song‐like vocalizations, analogous to those produced by birds.^64^ These protomusical utterances were, by hypothesis, free of any specific propositional meanings, just as instrumental music or birdsong are today. The crucial linguistic innovation linking these “songs” to meanings (requiring the independent innovation of meaningful words and sentences) occurred later in our evolutionary history.^133^, ^134^ This system, which by hypothesis had complex, learned vocal sequences but lacked propositional meaning, can be termed as “musical protolanguage” (although Darwin did not use this term).

If Darwin was correct, what structural properties might these early songs have had? Clearly, proto‐musical songs would have had “rhythm” in the sense that modern speech does,^135^ at a rate roughly shared by many nonhuman primate vocalizations.^109^, ^110^, ^114^, ^115^ Whether these earliest songs also had an isochronic beat cannot be determined. This suggests that the role of the medial system in generating a syllabic frame would have been present early, as suggested by MacNeilage, but in the different context of song. Because modern singing, particularly in nonlyrical styles like jazz scat singing, happily tolerates repetition in neighboring frames more than (adult) language, we can imagine that these earliest songs were syllabically repetitive “du du du” sequences, with superimposed melodies as in reduplicated babbling.

But crucially, it seems unlikely that the *pitch* identity of consecutive segments was identical since this would yield boring monotonous protosongs (very different from bird or whale song). Rather it is hypothesized that most syllables in a song varied in pitch. Thus, when singing a remembered song, even if the medial system provided a repetitive syllabic frame, there was still a need for a lateral motor system (particularly the laryngeal motor cortex) to program the fundamental frequencies (“pitch”) of the upcoming notes. I suggest that the neural circuitry that would be necessary for this simple one‐variable melodic programming could then have later been exapted to provide variegated syllables (phonetic content) as well. Although speculative, this hypothesis is consistent with both ontogenetic evidence (babbling) and the similarities and differences between modern song and speech. It can be tested (and contrasted with the toolmaking hypothesis) by examining the neural underpinnings of different song and speech stimuli.^135^

Whichever of these two possible precursors applied (and it could be that both were relevant), my suggestion is that phonological hierarchy, and particularly the free combinatoriality of segments and syllables it implies, provided a crucial step in the evolution of speech. Once the capacity for fixed‐depth hierarchy was in place, and supported some functionally useful system of vocal communication in our ancestors (whether as song and/or meaningful speech in some lexical protolanguage^3^), the neural circuitry underlying it would be subjected to stabilizing selection—it would become a reliably developing system. There would probably also be directional selection to support greater complexity (either to generate musical variety or to generate a larger lexicon). In any case, I suggest that this phonological hierarchical system itself provided the preadaptation that was exapted in the crucial bridge to phrasal syntax: the capacity for hierarchical embedding of greater depth. Such phrasal embedding could use much the same computational underpinnings as phonology, and would simply require that the stack (or more likely stacks) supported by Broca's region through its connections with the temporoparietal cortex be enlarged in capacity and scope, and that the stack could hold units above the word level. This could evolve via the “standard” duplicate‐and‐diverge processes discussed throughout this paper.

### Summary and comparison with previous models

Summarizing the argument above, I have highlighted the importance of the step from fixed rote sequences, learned verbatim through trial‐and‐error, to flexibly recombinant phonology. Although it may not be immediately obvious that hierarchy is needed for such flexibility, it is a matter of fact that hierarchical structure exists in modern phonology and that this modern system is freely combinatorial (at least in adults). I think that the sorts of memory constraints discussed by Miller,^136^ and the planning efficiency considerations necessary for complex action discussed by Simon^137^ probably both played a role.^10^ Once it originated, this limited phonological hierarchy would have supported a highly generative system, yielding a combinatorial explosion of possible words (and/or songs). But linking this signal‐generating system to the conceptual system of compositional meaning required further innovations, in particular the self‐embedding property of phrasal syntax. I suggest, based on the neural circuitry involved, that these further properties built upon (“exapted”) the more limited abilities of phonological syntax.

I am not the first to suggest that phonological hierarchy paved the way for later syntactic exaptations. Carstairs‐McCarthy^13^, ^138^ has proposed a multistage model in which an initial descent of the larynx led to phonological diversity, which due to synonymy avoidance led to a profusion of meaningful words. Keeping these straight required an innovation of syllabic structure, which in turn provided the raw material for phrasal syntax. My hypothesis here differs in several fundamental ways. First, I have argued that the descent of the larynx was immaterial and that preexisting primate vocal anatomy and lip‐smacking behavior were enough for an initial sequentially structured vocal communication system. Second, the crucial hierarchical aspect of phonology that I deem to be preadaptive was not the syllable‐internal structure posited by Carstairs‐McCarthy, but rather phonological phenomena that span multiple syllables including metrical stress patterns, rhyme, syllabic variegation, vowel harmony, and the like. For example, simply stipulating that “each syllable must be different” requires a one‐back memory of the last syllable, and phenomena like alliteration or rhyme require a working memory buffer to hold one syllable identity over an arbitrary time period until a matching syllable is found (or generated). These closely parallel related rhythmic phenomena found in music.^135^, ^139^

## Conclusions

In conclusion, Peter MacNeilage's FCT has received strong recent empirical support and should play an important role in future theorizing about the evolution of speech, and particularly phonological structure, and the relationship of these to other aspects of language like syntax and semantics. Although it is certainly possible that the evolution of hierarchy in phonology, syntax, semantics, and music occurred independently, it seems more parsimonious to pursue a model in which they share computational properties, neural machinery, and evolutionary history.^9^, ^10^ In this paper, I have outlined such a model, and some of its predictions, but even if this model turns out to be incorrect in detail, I hope that the overall approach inspires future, better models in a similar spirit.

## Competing interests

The author declares no competing interests.
